# High Compression Performance and Energy Absorption of Wood-Based Grid Sandwich Structure with Jute Fabric/Epoxy Composite Core

**DOI:** 10.3390/polym18141753

**Published:** 2026-07-17

**Authors:** Xue Wang, Hanxiang Guo, Xiaohong Yu

**Affiliations:** 1 College of Chemistry and Materials Engineering, Zhejiang A&F University, Hangzhou 311300, China; 2 Bamboo Industry Institute, Zhejiang A&F University, Hangzhou 311300, China; guohx2026@163.com

**Keywords:** sandwich structure, jute fabric, compression behavior, energy absorption, failure mechanism

## Abstract

The wood-based grid sandwich structure with a high load-to-mass ratio and specific strength was prepared with the core of KH-560-modified jute (*Corchoruscapsularis*) fabric-reinforced epoxy laminated composite (JFRELC). The compressing behavior and energy absorption characteristics of pure grid cores (GC50#, GC80#) and grid sandwich structures (GS50#, GS80#) were analyzed and compared. The failure mechanism of the fracture surfaces of jute fabrics of grid sandwich cores was clarified by SEM. The results showed that the core made of JFRELC-80# had a good performance for the grid sandwich structure by tenon-and-mortise linking. The load-bearing capacity and energy absorption performance of this wood-based grid sandwich structure can be comparable to that of some glass and carbon fiber reinforced composite sandwich structures, and even show certain advantages. The failure modes of the grid sandwich structure were panel cracking, core buckling and core collapse. The failure mechanisms of jute fabrics in epoxy resin were fiber pull-out and fiber splitting.

## 1. Introduction

The composite sandwich structure is a lightweight and high-strength structural material, which can be composed of panels and periodically arranged cores. Typical sandwich structures include honeycomb sandwich structure [1,2], corrugated sandwich structure [3,4,5] and lattice sandwich structure [6,7]. Carbon fiber or glass fiber-reinforced composite sandwich structures have been widely used in aerospace, shipbuilding, automobile manufacturing and other fields [8,9,10,11]. Reducing carbon emissions and sustainable development are the consensus of the industry. The wood-based composite sandwich structure has the characteristics of lightweight, heat insulation, noise reduction, environmental protection and sustainability. Therefore, using renewable natural fibers to manufacture lightweight and high-strength structural engineering materials has great significance and application prospects [12,13,14].

The compressive behavior of sandwich structures is an important load-bearing property. The panel type and core material affect the compressive strength, stiffness and energy absorption of the sandwich structure. Studies have shown that cardboard was significantly stiffer than polyethylene terephthalate glycol (PETG) board, and the cardboard panel showed a high ability to absorb energy [15]. The lattice sandwich structure with the inclination angle of the core cell ribs of *φ_x_* = *φ_y_* = 65° had the highest compressive strength [16]. The failure modes of wood-based sandwich structure were panel cracking, core buckling, core shear failure or core collapse [4,17,18,19]. The sandwich structure with a rod-shaped lattice core was prone to core buckling and core shear failure [20,21,22]. It is worth noting that the core layer is the most designable part of the sandwich structure, and it also affects the overall stability and bearing capacity of the sandwich structure. So, the development of core materials with high strength and dimensional stability is one of the keys to fabricating lightweight and high-strength wood-based sandwich structures.

Jute fiber, as the second largest plant fiber next to cotton fiber, has good mechanical properties, lightweight, abundant resources, low price and environmentally friendly, which are suitable for the preparation of the core of wood-based sandwich structure [23,24,25,26]. KH-560-modified jute fabric-reinforced epoxy resin composite exhibited good mechanical properties when applied to wood-based sandwich beams [12]. In addition, the interface connection between the panel and core impacted the bearing capacity and thermal stability of the sandwich structure [27,28]. The interlocking connection avoided the core pulling out from the panel, and the continuous core was conducive to enhancing the stability of the structure. The compression behavior and energy absorption characteristics of a wood-based sandwich structure with the jute fabric-reinforced epoxy grid core have not been evaluated.

Therefore, in order to further improve the properties of lightweight, high-strength and energy absorption of the wood-based sandwich structure, in this paper, using KH-560-modified jute fabric-reinforced epoxy laminated composite (JFRELC) as the core and spruce wood as the panel. A kind of tenon-and-mortise linking was used as the connection between core sheets and panels. The influence mechanism of different yarn densities of jute fabrics on the compressing behavior and energy absorption characteristics of the sandwich structure was studied. The evaluation mechanism of the KH-560-modified jute fabric-reinforced epoxy resin composite applied to the wood-based sandwich structure was revealed, and the failure mechanism was elucidated. This study provides a theoretical basis for the development of wood-based sandwich structures with a high load-mass ratio and extends the application range of green plant fiber composites.

## 2. Materials and Production

### 2.1. Materials

In this paper, the grid sandwich structure included a panel and a core, which were different. The spruce wood was used as the panel of the grid sandwich structure, and it was purchased from Jilin Forest Industry Group Co., Ltd. (Changchun, China). The spruce panel had properties of lightweight, easy processing and excellent mechanical properties, with a density of 0.43 g/cm^3^ and a moisture content of 14%. KH-560-modified jute fabrics-reinforced epoxy resin laminated composites (JFRELCs) were used as the core for the grid sandwich structure. The plain weave jute fabrics with the yarn density of 50/10 cm and 80/10 cm were the reinforcement of the core material, which were purchased from Guangzhou Yin Fan Textile Limited (Guangzhou, China). The meaning of 50/10 cm of yarn density was 50 yarns per 10 cm, which was represented by 50#. And 80/10 cm had the same meaning and was represented by 80#. Epoxy resin 6101, polyamide resin 650 and diluent 692 were provided by Zhenjiang Danbao Resin Co., Ltd. (Zhenjiang, China). The Silane coupling agent (KH-560) and glacial acetic acid were supplied by Harbin Sailebo Technology Ltd. (Harbin, China).

### 2.2. Production of Grid Sandwich Structures

**Figure 1:**
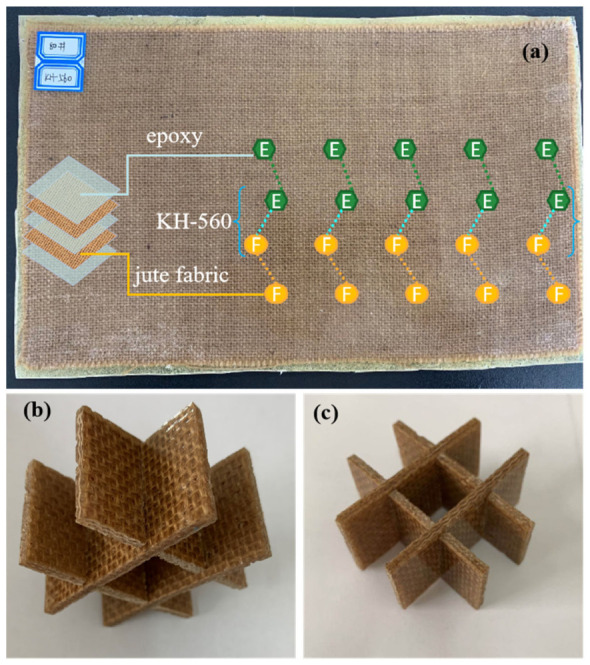
The samples of (**a**) KH-560-modified jute fabrics reinforced epoxy laminated composite (JFRELC), (**b**,**c**) the grid cores composed of JFRELC.

The production of grid sandwich structures mainly included two aspects: one was the preparation of the grid cores, and the other was the assembly of panels and grid cores. In order to enhance the lightweight and high strength of the sandwich structure, the grid core was made of two layers of jute fabrics reinforced epoxy composites (JFRELCs) (Figure 1a), and KH-560 grafting treatment was used to improve the interface integration of JFRELCs [12]. Epoxy resin 6101, polyamide resin 650 and diluent 692 were the matrix materials of JFRELCs, mixed in a ratio of 6101:650:692 = 10:6:1. The compression samples of grid cores (GCs) with yarn densities of 50# and 80# reinforced composites were carried out for fully understanding the compressive bearing capacity and failure mode of the core layer, as shown in Figure 1b,c. The length, width, and height of the core sheets of GCs were 57 mm, 3 mm and 30 mm, respectively, and the groove depth, groove width and interval were 15 mm, 3 mm and 17 mm, respectively.

**Figure 2:**
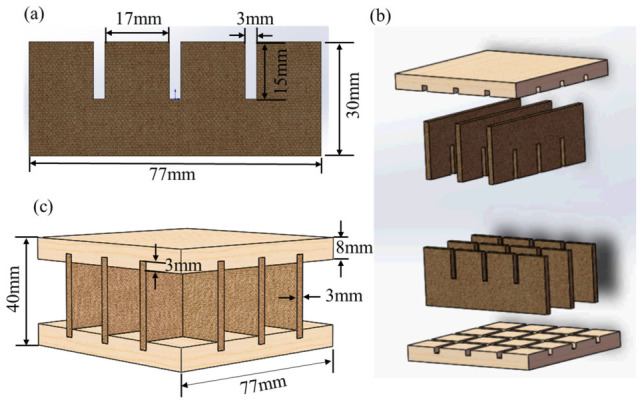
Wood-based grid sandwich structure: (**a**) the sheet of core layer; (**b**) assembly of sandwich structure by tenon-and-mortise linking; (**c**) the geometry of the sandwich structure.

JFRELCs were prepared and sawn into serrated sheets as cores of grid sandwich structures, which were assembled with spruce panels (Figure 2a). The connection between core sheets and panels was a kind of tenon-and-mortise linking (Figure 2b). The geometric dimensions of grid sandwich structures were designed according to GB/T 1453-2005 [29] for the flat-compression test, as shown in Figure 2c. The size of compression specimens of the grid sandwich structure (GS) was 77 mm × 77 mm × 40 mm. The thickness of the panel and core layer was 8 mm and 24 mm, respectively. The thickness and the groove width of the core sheets were both 3 mm, the groove depth was 15 mm, and the notch spacing was 17 mm (Figure 2a).

## 3. Methods and Testing

### 3.1. The Relative Density of Grid Sandwich Structure

**Figure 3:**
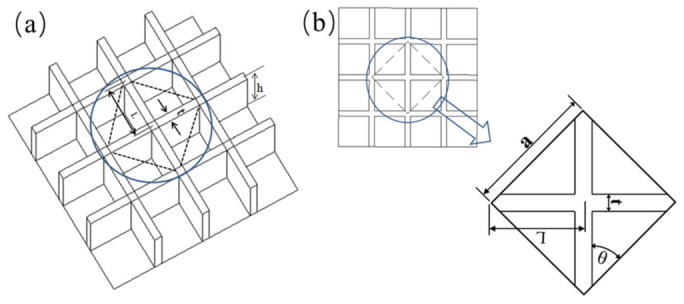
Representative unit of the grid sandwich structure: (**a**) sandwich structure model; (**b**) schematic diagram of the planar geometry of the representative unit.

For the convenience of calculation, take a representative unit in the core layer of the grid sandwich structure and mark its geometric relationship (Figure 3a,b), where *L* = *a*·*cosθ*. The equivalent density *ρ_c_* of the sandwich structure means that the sandwich structure is equivalent to a piece of homogeneous material of equal volume, and the ratio of the mass to volume of this material is the equivalent density of the sandwich structure. The relative density *ρ_h_* of the unit core of the sandwich structure is the ratio of the equivalent density *ρ_c_* to the true density *ρ*_0_. The *ρ_h_*, *ρ_c_* and *ρ*_0_ of the grid core were derived as (1), (2) and (3):

(1) $$\rho_{h}=\frac{\rho_{c}}{\rho_{0}}=\frac{2t}{L^{2}}\left(2L-t\right)\cos^{2}\theta$$

(2) $$\rho_{c}=\frac{m}{a^{2}h}$$

(3) $$\rho_{0}=\frac{m}{\left(4Lt-2t^{2}\right)h}$$

where *L* and *t* are the length and thickness of the sheet wall of the unit core of the grid sandwich structure. The *m*, *a* and *h* represent the mass, length and height of the unit core of the grid sandwich structure, respectively. *θ* is the angle between the sheet wall of the unit core and the boundary of the unit core.

### 3.2. Compression Performance of Grid Sandwich Structure and Core

**Figure 4:**
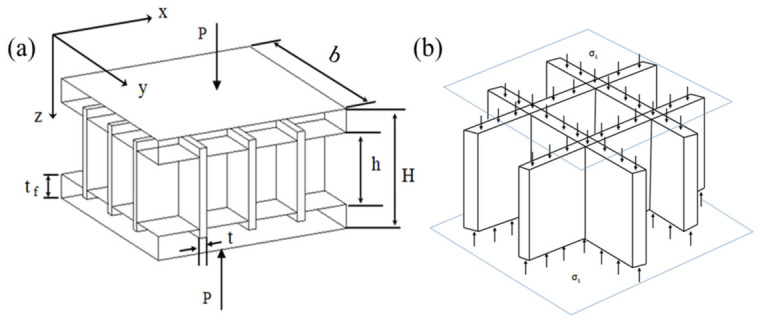
Schematic diagram of stress: (**a**) overall sandwich structure, (**b**) the wall of the core.

Figure 4 is the schematic diagram of the load-bearing capacity of the overall grid sandwich structure and the grid wall of the core. Both the sandwich structure and the core are deformed under the action of the Z-direction bearing capacity *P*. The relevant compressive mechanical properties were calculated with reference to the standard GB/T 1453-2005 [29].

(4) $$\sigma_{c}=\frac{P}{b^{2}}$$

(5) $$E_{c}=\frac{\Delta P\left(H-2t_{f}\right)}{\Delta h\cdot b^{2}}$$

where *σ_c_* and *E_c_* are the compression strength and compression elasticity modulus of the grid sandwich structure, respectively. The *P*, *b*, *H*, and *t_f_* represent the compression failure load, length of sandwich structure, height of sandwich structure, and thickness of panel, respectively. Δ*h* is the deformation increment corresponding to the elastic phase’s load increment Δ*P*.

(6) $$\sigma_{s}=\frac{4P}{4nLt-nt^{2}}$$

(7) $$E_{s}=\frac{\Delta P\cdot h_{c}}{\left(nLt-\frac{n}{4}t^{2}\right)\Delta h}$$

where *σ_s_* and *E_s_* are the compression strength and compression elasticity modulus of the core wall, respectively. The *n* and *h_c_* are the number of core walls and the thickness of the sample core, respectively.

In addition to the above flat pressure performance indicators, the specific strength *σ_cq_* is an equally important indicator for the sandwich structure. The specific strength can evaluate whether the material is light and strong. The greater the specific strength, the lighter and stronger the material. *σ_cq_* is the ratio of the material’s compression strength *σ_c_* to the apparent density *ρ_cq_*, which is the ratio of the material’s mass to the apparent volume, and the apparent volume is the sum of the material’s actual volume and the volume of the closed pores. *σ_sq_* and *ρ_sq_* are the specific strength and the apparent density of the grid core, respectively.

(8) $$\sigma_{cq}=\frac{\sigma_{c}}{\rho_{cq}}$$

(9) $$\sigma_{sq}=\frac{\sigma_{s}}{\rho_{sq}}$$

### 3.3. The Energy Absorption Performance of Grid Sandwich Structures and Cores

The grid sandwich structure has a good buffering effect and energy absorption capacity due to its reasonable hollow structure. In order to evaluate the energy absorption capacity of the grid sandwich structure, the total energy absorption *E_A_*, specific energy absorption *ξ_EA_*, average load *P_m_* and load efficiency *A_E_* are evaluated for the flat pressure energy absorption performance of the grid sandwich structures and the grid cores. The energy absorption of the sandwich structure during the deformation process under the action of external force is the total energy absorption [30], and the area between the load–displacement curve and the X-axis can be visually expressed as the total energy absorption. The relevant formulae are as follows:

(10) $$E_{A}=\int_{0}^{\delta }P\left(x\right)dx$$

(11) $$\xi_{EA}=E_{A}/m$$

(12) $$P_{m}=E_{A}/\delta$$

(13) $$A_{E}=P_{m}/P_{k}$$

where *m*, *δ* and *P_k_* are the quality, deformation and peak load of grid sandwich structures and grid cores, respectively.

### 3.4. Compressive Tests of Panel Materials and JFRELCs

The compressive performance of the spruce wood with perpendicular to grain (SW-X) and parallel grain (SW-Y) was tested with reference to GB/T 1939-2009 [31] and GB/T 1935-2009 [32]. The size of the sample was 30 mm × 20 mm × 20 mm, and the length direction was the wood along the grain. Ten samples were taken to determine the compressive strength of the spruce wood with perpendicular to grain and parallel grain, respectively, and average values were calculated.

The compressive strength of the before and after the KH-560-modified JFRELCs with 50# and 80# jute fabrics reinforced were tested according to GB/T 1448-2005 [33]. The sample size was 25 mm × 10 mm × 10 mm; there were 10 samples for each group testing, and the average values were reported.

### 3.5. Compressive Performance Tests of Grid Sandwich Structure and Core

**Figure 5:**
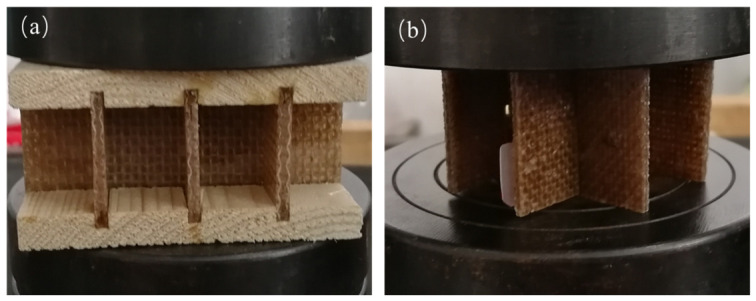
Device for compression test of (**a**) the grid sandwich structure and (**b**) the grid core.

As shown in Figure 5, the compressive properties of grid sandwich structures (Figure 5a) and grid cores (Figure 5b) were tested on the universal testing machine CMT5504 to analyze the failure modes and failure mechanism, the geometric design of sandwich structures and cores. The flat compression test was carried out with reference to GB/T 1453-2005 [29]. The flat compression sample size of the grid sandwich structure and grid cores were 77 mm × 77 mm × 40 mm and 57 mm × 57 mm × 30 mm, respectively. Each sample consisted of two groups, each group had 5 samples for the flat compression performance test, and average values were calculated. Figure 5a shows the sample of jute fabric-reinforced grid sandwich structure with a yarn density of 50#; Figure 5b shows the grid core sample of the jute fabric reinforced with a yarn density of 80#. They were loaded at a speed of 0.5 mm/min; the jute fabrics (50#, 80#) were modified with KH-560 to improve the interfacial compatibility between the jute fabric and epoxy resin.

### 3.6. SEM Analysis of Fracture Surfaces of Grid Sandwich Cores

Scanning electron microscopy (SEM) (Quanta 200, FEI Co., Hillsboro, NH, USA) was used to observe the failure mechanism of the fracture surface of the grid sandwich structure core. Samples were taken from the cores destroyed by compression testing and coated with a thin layer of gold to observe.

## 4. Results and Discussion

### 4.1. Compressive Performance Analysis of Spruce Wood and JFRELCs

**Figure 6:**
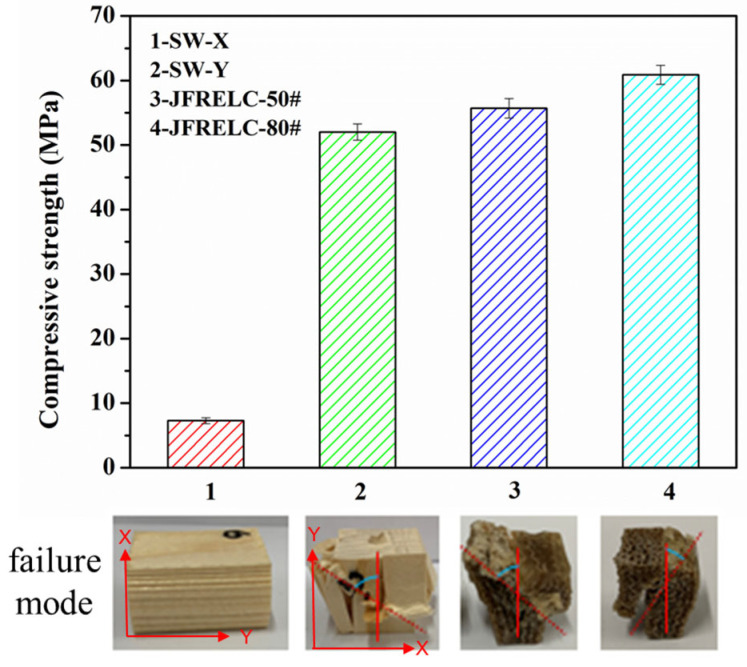
Compressive strength and failure mode of spruce wood (SW-X, SW-Y) and KH-560-modified jute fabric/epoxy laminate composites (JFRELC-50#, JFRELC-80#).

Figure 6 shows the compressive strength and failure modes of the panel and core materials of the grid sandwich structure. The compressive strength of spruce wood with perpendicular to grain (SW-X) and parallel grain (SW-Y) is 7.28 ± 0.45 MPa and 52.01 ± 1.26 MPa, respectively. Generally speaking, the compressive strength of the wood with perpendicular grain is 10% to 20% of the wood with parallel grain, and the experimental data is within this range. JFRELC-50# and JFRELC-80# have higher compressive strength than SW-Y, and JFRELC-80# has higher strength (60.89 ± 1.47 MPa) than JFRELC-50# (55.69 ± 1.51 MPa) because it has a higher fabric density. The KH-560 modification forms the Si-O-C bond between the jute fiber and epoxy, which is equivalent to the “bond bridge” that organically combines the epoxy and the jute fabrics [24], thereby improving the interface compatibility of JFRELCs.

In terms of failure modes, the cell wall of SW-X is compressed to form the layered stacked structure, which becomes compressed wood. SW-Y, JFRELC-50# and JFRELC-80# are unstable and have a split failure. The inclination angle of fiber fracture during compression depends on the density of the material; the higher the density, the smaller the inclination angle. It can be seen from the Figure 6 failure mode that ∠SW-Y is greater than ∠JFRELC-50# and greater than ∠JFRELC-80#, because the density of spruce, 0.43 g/cm^3^, is less than 0.90 g/cm^3^ (JFRELC-50#) and less than 1.09 g/cm^3^ (JFRELC-80#). Therefore, the KH-560-modified JFRELC is suitable as the core material of the wood-based grid sandwich structure.

### 4.2. Compression Failure Behavior Analysis of Grid Sandwich Structure and Core

**Figure 7:**
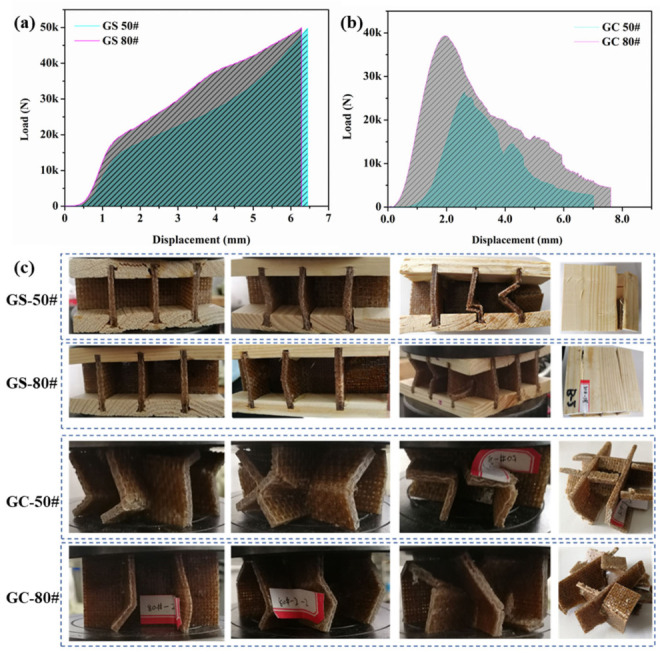
Compression load–displacement curves of (**a**) grid sandwich structures (GS 50#, GS 80#), (**b**) grid cores (GC 50#, GC80#), and (**c**) the failure modes of grid sandwich structures (GS 50#, GS 80#) and grid cores (GC 50#, GC80#).

Figure 7 shows the compression behaviors of grid sandwich structures (GS50#, GS80#) and grid cores (GC50#, GC80#). With the increase in loads, the load–displacement curves of GS50# and GS80# present the elastic stage and an elastic–plastic stage (Figure 7a). Under the initial load, the curve has a gentle inelastic stage, which is due to the slight error in the dimensions of the panel and core of the sandwich structure during processing, resulting in a gap between the panel and the core. When the displacement reaches about 0.5 mm, the curve begins to enter the elastic rise stage. Combined with the failure modes of GS50# and GS80# in Figure 7c, the spruce panels of the sandwich structure show indentation failure, and the core layer has no obvious deformation in the elastic stage. With the increase in the load, in the elastic–plastic stage, the core layer of the sandwich structure undergoes compressive buckling deformation, until the core is finally compressed and yields failure, and the panel splits along the grain direction of the wood. The final bearing capacity of the sandwich structure (GS 80#) is as high as 50 KN, which is 26.8% higher than that of GC 80#, and the total deformation of the sandwich structure is about 6–7 mm.

Figure 7b shows the load–displacement curves of the grid cores (GC50#, GC80#). Under the initial load, the load–displacement curves of the grid cores also have a flat phase, and the reason is the same as Figure 7a, combined with the failure modes of GC50# and GC80# in Figure 7c. In the linear elastic stage (GC50#: 0–25 KN, GC80#: 0–40 KN), the cracks are formed at the grooves of the grid walls of GC50# and GC80#. With the increase in loads, the cracks expand until the final compressive buckling failure, and then curves enter the descending stage. The total deformation of the square core is about 7 mm~8 mm.

### 4.3. Compression Bearing Capacity Analysis of Grid Sandwich Structure and Core

**Figure 8:**
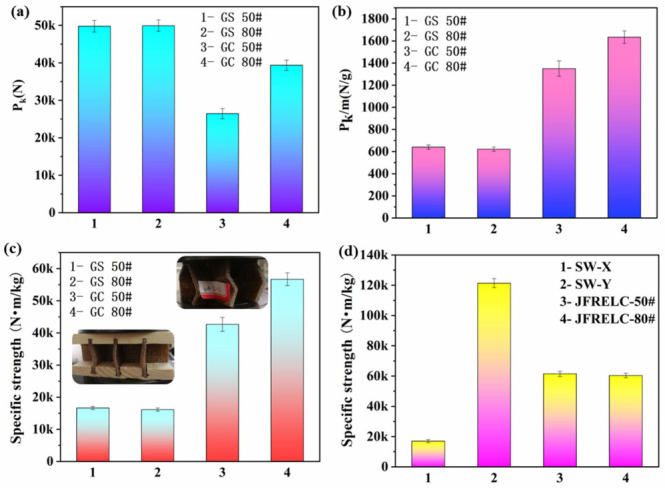
Histograms of grid sandwich structures (GS 50#, GS80#) and grid cores (GC 50#, GC 80#): (**a**) maximum load-carrying capacity (P_k_), (**b**) load-to-mass ratio (P_k_/m), (**c**) specific strength, (**d**) specific strength of spruce wood (SW-X, SW-Y) and JFRELCs (JFRELC-50#, JFRELC-80#).

Figure 8a shows that the maximum loads of GS 50#, GS 80#, GC 50# and GC 80# are 49.797 ± 1.564 KN, 49.946 ± 1.542 KN, 26.461 ± 1.358 KN and 39.387 ± 1.369 KN, respectively. Figure 8b shows that the load-to-mass ratios of GS 50#, GS 80#, GC 50# and GC 80# are 640.889 ± 20.13 N/g, 621.997 ± 19.21 N/g, 1350.065 ± 69.28 N/g and 1634.296 ± 56.83 N/g. It can be seen that the load-to-mass ratios of GC 50# and GC80# are higher than those of GS 50# and GS 80#. The load-to-mass ratio reported for pineapple leaf fiber reinforced structures was 79.323 N/g [34], while GC80# achieved a value approximately twenty times higher (Table 1). The specific strengths of GS 50#, GS 80#, GC 50# and GC 80# are 16.639 ± 0.515 KN·m/kg, 16.148 ± 0.498 KN·m/kg, 42.668 ± 2.173 KN·m/kg and 56.702 ± 1.963 KN·m/kg, respectively (Figure 8c). The specific strength of GC 50# is 156.4% higher than that of GS 50#, and the specific strength of GC 80# is 251.1% higher than that of GS 80#. Compared with the existing related research (Table 1), the specific strength of the carbon fiber/epoxy composite pyramid sandwich structure was 13.69 KN·m/kg [35]. The specific strength of the sandwich structure with wood–plastic composite as the panel and carbon fiber/epoxy composite as the core was between 3.814 KN·m/kg and 30.417 KN·m/kg [21]. Figure 8d shows that the specific strength of SW-X is 16.993 ± 1.05 KN·m/kg, which is close to that of GS 50# and GS 60#, while the specific strength of JFRELC is higher, about 60 KN·m/kg. The results indicate that the grid sandwich structure with JFRELC core has the performance advantages of lightweight and high strength.

Table 2 shows the compression properties of grid sandwich structures (GS 50#, GS80#) and grid cores (GC 50#, GC 80#). The compressive strength of the lattice sandwich structure reinforced with glass fiber was 5.587 MPa (Table 1) [36], and GS50# and GS80# are 50.3% and 50.8% higher than that of them, respectively. The flat compressive strength of the carbon fiber/epoxy resin pyramid sandwich structure prepared by water cutting process was between 2.40 MPa and 2.95 MPa [37], and GS50# and GS80# are about 184.7~249.9% and 185.5~251.0% higher than that of them, respectively. And the compressive strength of GC 50# and GC 80# is better than GS50# and GS80#, because of the excellent mechanical strength of the JFRELC core. The results suggest that the compressive bearing capacity of GS50# and GS80# can be comparable to that of some glass and carbon fiber reinforced composite sandwich structures, and even shows certain advantages.

### 4.4. Energy Absorption Performance Analysis of Grid Sandwich Structure and Core

**Figure 9:**
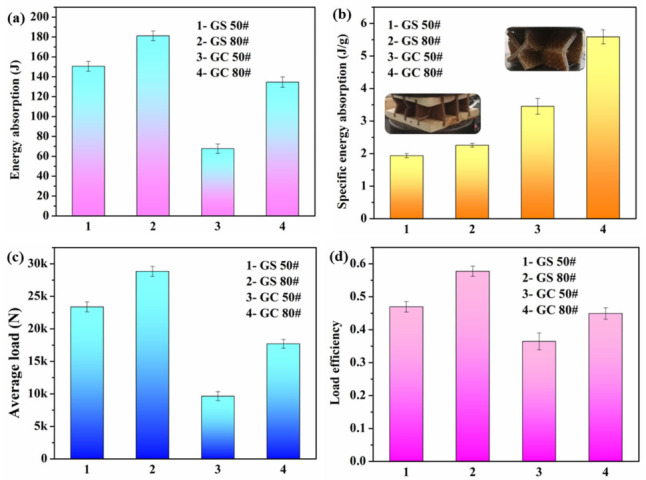
Histograms of grid sandwich structures (GS 50#, GS80#) and grid cores (GC 50#, GC 80#): (**a**) energy absorption, (**b**) specific energy absorption, (**c**) average load, (**d**) load efficiency.

Figure 9 shows the energy absorption performance of the grid sandwich structure and the grid core calculated according to Formulae (10)~(13). The total energy absorption values of GS 50#, GS 80#, GC 50# and GC 80# are 150.583 ± 5.04 J, 181.211 ± 4.85 J, 67.698 ± 4.77 J and 134.631 ± 5.21 J, respectively (Figure 9a). The bearing capacity and specific strength of GS 50# and GS 80# have no obvious effect (Figure 8a–c), but the total energy absorption of GS 80# is 20.3% higher than that of GS 50#. This is also reflected in the load–displacement curve (Figure 7a). It proves that the bearing capacity of the grid sandwich structure is determined by the synergistic effect of the panel and the core, and the increase in the yarn density of the jute fabrics of the core is beneficial to improve the energy absorption performance of the grid sandwich structure. Differences in fabric yarn density can affect the resin distribution inside the composite and its local stiffness, and also impact the ability to absorb energy and mechanical performance.

Figure 9b shows that the specific energy absorption of GS 50#, GS 80#, GC 50# and GC 80# are 1.938 ± 0.065 J/g, 2.257 ± 0.060 J/g, 3.454 ± 0.243 J/g and 5.586 ± 0.216 J/g, respectively. The specific energy absorption of GS80# and GC80# is 16.5% and 61.7% higher than that of GS50# and GC50#, respectively. Although the total energy absorption of GS is higher than that of the grid core, the specific energy absorption, load-to-mass ratios and specific strengths of GCs are higher than those of the grid sandwich structure. Because wood is anisotropic, the wood fiber properties along the grain are much stronger than across the grain. During compression, the transverse fibers of the wooden panels of GSs were prone to cracking. The GC was prepared with mesh-like jute fabric-reinforced epoxy resin, which overcame some of the anisotropy. Therefore, in practical applications, for sandwich structures that do not require panels, GCs can be considered.

Figure 9c shows that the average loads of GS 50#, GS 80#, GC 50# and GC 80# are 23.376 ± 0.782 KN, 28.841 ± 0.771 KN, 9.645 ± 0.679 KN, and 17.698 ± 0.685 KN, respectively. It can be seen that the average load of GS 80# is the largest, which is 23.4% higher than that of GC 50#. The average load of GC 80# is 83.5% higher than that of GC 50#. The load efficiencies of GS 50#, GS 80#, GC 50# and GC 80# are 0.469 ± 0.016, 0.577 ± 0.015, 0.364 ± 0.026 and 0.449 ± 0.017, respectively (Figure 9d). The maximum specific energy absorption of the sandwich structure was 1 J/g (Table 1) [38]. And the specific energy absorption of the sandwich structure with glass fiber-reinforced composite sheet and coconut core was 1.0 J/g, which was higher than that of the sandwich structure with carbon fiber reinforced composite sheet and coconut core (0.91 J/g) [30], and GS 80# is about two times that. Overall, the energy absorption characteristics of GS80# are excellent. From the load–displacement curve (Figure 7), it can be seen that during the elastic stage, GS mainly absorbs energy through the compressive deformation of the wooden panel. When cracks appear in the groove area of the panel, the load–displacement curve enters the elastoplastic stage. As the load continues to increase, the grid core material begins to buckle and deform, absorbing energy until it is crushed. However, the natural fiber-reinforced composite sandwich structure has limitations in moisture absorption, durability, aging resistance, and long-term structural stability. These limitations should be provided with a balanced evaluation in engineering applications.

### 4.5. SEM Analysis of Fracture Surfaces of Grid Sandwich Cores

**Figure 10:**
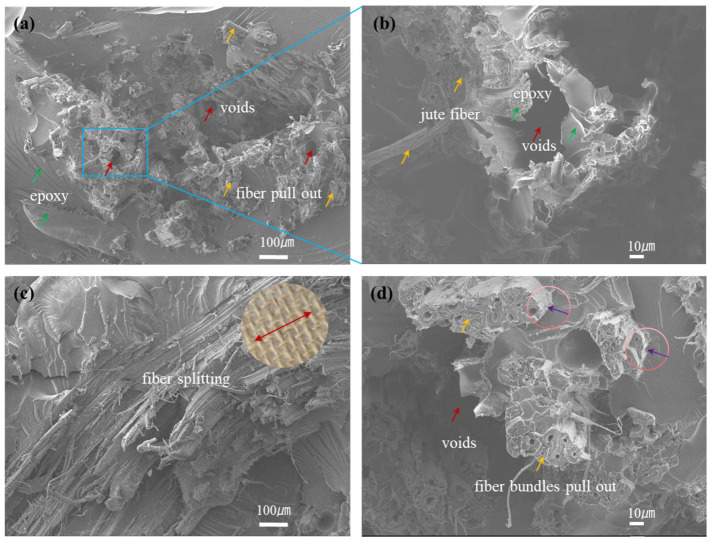
SEM images of fracture surfaces of grid sandwich cores: (**a**) the transverse fibers of jute fabric of grid cores, (**b**) the partial enlargement of (**a**), (**c**) the longitudinal fibers of jute fabric of grid cores, (**d**) the enlarged pull-out jute fiber bundles of grid cores.

Figure 10 is the SEM images of fracture surfaces of grid sandwich cores from which samples were taken from the cores destroyed by compression testing. The observation results show that the failure modes of jute fabrics in epoxy resin are fiber pull-out and fiber splitting. The pull-out fibers and splitting fibers are mainly the jute fiber bundles in the direction and perpendicular to the direction of force, respectively (Figure 10a,c). The cross-intersecting jute fabric enhances the mechanical properties in both directions. Figure 10b is a partial enlargement of Figure 10a, which can be seen that there are voids left after the fiber bundles pulled out, and there is remaining epoxy resin on the protruding fibers, indicating that the fiber bundles of the jute fabric treated with KH-560 can be effectively bonded with the epoxy resin. Figure 10d further shows that the pull-out fibers exhibit a clean-cut fracture surface, and the interface between the fiber outer edge and the resin matrix is well bonded. From the SEM analysis, the macroscopic and microscopic failure mechanisms of the sandwich structure are further elucidated.

## 5. Conclusions

The grid sandwich structures were prepared with KH-560-treated jute fabric/epoxy laminated composite core and the spruce panel by tenon-and-mortise linking. The load-bearing capacity and energy absorption performance of the sandwich structure can be comparable to that of glass fiber and carbon fiber composite sandwich structures, and even show certain advantages.

(1) Considering all aspects, the compression failure behavior, bearing capacity and energy absorption performance of GS 80# are the best. The maximum failure load, load-mass ratio, specific strength and total energy absorption of GS 80# are 49.946 ± 1.542 KN, 621.997 ± 19.21 N/g, 16.148 ± 0.498 KN·m/kg and 181.211 ± 4.85 J, respectively. The total energy absorption, specific energy absorption, average load and load efficiency of GS80# are 20.3%, 16.5%, 23.4% and 23% higher than those of GS 50#, respectively.

(2) The flat compression failure modes of the grid sandwich structures are core buckling, core collapse and panel cracking. The failure modes of the grid cores are that the core lattice wall buckled at first, cracks formed at the grooves of the lattice walls, and, finally, the core collapsed. The dominant failure mechanisms for the jute fabric/epoxy composites are fiber pull-out and fiber splitting.

(3) The sandwich structure exhibited high compressive bearing and energy absorption performance, and further research on its impact resistance, cyclic compression behavior, fatigue resistance, or environmental durability will be conducted.

## Figures and Tables

**Table 1:** Comparative analysis of compression performance of GS 50#, GS 80#, GC 50#, and GC 80# with published studies.

Samples	Load-to-Mass Ratio (N/g)	Specific Strength *σ_cq_* (KN·m/kg)	Compressive Strength *σ_c_* (MPa)	Specific Energy Absorption *ξ_EA_* (J/g)
GS 50#	640.889 ± 20.13	16.639 ± 0.515	8.399 ± 0.20	1.938 ± 0.065
GS 80#	621.997 ± 19.21	16.148 ± 0.498	8.424 ± 0.26	2.257 ± 0.060
GC 50#	1350.065 ± 69.28	42.668 ± 2.173	--	3.454 ± 0.243
GC 80#	1634.296 ± 56.83	56.702 ± 1.963	--	5.586 ± 0.216
pineapple leaf fiber-reinforced [34]	79.323	--	--	--
carbon fiber-reinforced [35]	--	13.69	--	--
carbon fiber-reinforced [21]	--	3.814~30.417	--	--
glass fiber-reinforced [36]	--	--	5.587	--
carbon fiber-reinforced [37]	--	--	2.40~2.95	--
PET (poly-ethylene terephthalate) [38]	--	--	--	1.0
glass fiber and carbon fiber-reinforced [30]	--	--	--	1.0 and 0.91

**Table 2:** Compression properties of grid sandwich structures (GS 50#, GS 80#) and grid cores (GC 50#, GC 80#).

Samples	Relative Density *ρ_h_*	The Grid Sandwich Structure		The Core Wall	
		Compression Strength *σ_c_* (MPa)	Compression Elasticity Modulus *E_c_* (MPa)	Compression Strength *σ_s_* (MPa)	Compression Elasticity Modulus *E_s_* (MPa)
GS 50#	0.272	8.399 ± 0.26	55.243 ± 1.42	36.754 ± 1.15	241.743 ± 4.12
GS 80#	0.272	8.424 ± 0.26	92.969 ± 1.52	36.743 ± 1.13	405.501 ± 5.69
GC 50#	0.274	--	--	38.680 ± 1.97	904.027 ± 5.82
GC 80#	0.276	--	--	57.201 ± 1.98	1362.164 ± 6.78

## Data Availability

The original contributions presented in this study are included in the article. Further inquiries can be directed to the corresponding authors.
